# Prevalence of Osteosarcopenic Adiposity in Apparently Healthy Adults and Appraisal of Age, Sex, and Ethnic Differences

**DOI:** 10.3390/jpm14080782

**Published:** 2024-07-23

**Authors:** Selma Cvijetić, Irena Keser, Dario Boschiero, Jasminka Z. Ilich

**Affiliations:** 1 Division of Occupational and Environmental Health, Institute for Medical Research and Occupational Health, Ksaverska cesta 2, 10000 Zagreb, Croatia; 2 Laboratory for Nutrition Science, Faculty of Food Technology and Biotechnology, University of Zagreb, Pierottijeva 6, 10000 Zagreb, Croatia; irena.keser@pbf.unizg.hr; 3 BioTekna, Marcon, 30020 Venice, Italy; dario.boschiero@biotekna.com; 4 Institute for Successful Longevity, Florida State University, 1107 West Call Street, Tallahassee, FL 32306, USA; jilichernst@fsu.edu

**Keywords:** osteosarcopenic adiposity, osteosarcopenic obesity, body composition, osteopenia, osteoporosis, sarcopenia, adiposity, obesity

## Abstract

Osteosarcopenic adiposity (OSA) syndrome, the coexistence of osteoporosis, sarcopenia, and adiposity (either excess or redistributed/infiltrated), has been studied globally in different populations and regions (mostly in East Asia, less in Europe and North America), resulting in varied prevalence. We aimed to determine the prevalence of OSA in a large population of apparently healthy Caucasian adults (18–90 years) and to compare it with the prevalence reported in other studies and other ethnicities. This study included 9719 participants (6412 women and 3307 men), stratified into four age-group categories, and recruited from the general medical practices in Italy. OSA was defined based on body composition measurements using bioelectrical impedance BIA-ACC^®^, which enables assessment of total bone mass, muscle/lean, and adipose tissues. The overall prevalence of OSA was 21.9% in women and 14.0% in men, and it significantly increased in every subsequent age group for both women and men (*p* < 0.001). The OSA prevalence was not significantly different between men and women below 40 years; however, it was considerably higher in women over 40 years. Participants with OSA had a significantly lower BMI compared to those without OSA, indicating OSA is a separate disorder not necessarily related to physiological fluctuations of BMI. The prevalence in Asian populations was lower than in our sample, indicating ethnic specificity. The relatively high prevalence of OSA detected in this study’s population across the age groups suggests the necessity for its appropriate and timely identification to prevent possible clinical outcomes, including fracture, dismobility, frailty, or chronic diseases.

## 1. Introduction

The changes in the main components of body composition, namely, bone, muscle, and adipose tissue, play a significant role in overall health [1,2], as well as in the onset and manifestations of chronic diseases [3]. The unfavorable changes in each tissue lead to osteopenia/osteoporosis, sarcopenia/dynapenia, and increased/redistributed adiposity, respectively. A coexistence of all three conditions is known as osteosarcopenic adiposity/obesity (OSA/OSO) syndrome, identified in 2014 [4]. Research on OSA has been conducted across the world in various populations, as recently reviewed [5,6,7,8]. The evidence points to the OSA syndrome being a serious body composition impairment that has a bidirectional relationship with cardiometabolic impairments (e.g., dyslipidemia, hypertension, increased inflammatory markers) [8] and possibly with some cancers [9], as well as several chronic diseases, including Cushing’s disease and kidney and liver diseases [10,11,12]. Of note is that the researchers in these studies employed different methodologies to identify OSA, which resulted in a wide range of reported prevalence, from <1% to ~90% [7]. This is an unprecedented range of prevalence for any condition but could be rationalized when considering factors that shaped the OSA identification: (a) utilization of various technologies with different diagnostic criteria and cutoffs; (b) diverse populations and geographic regions where the studies were conducted; and (c) a wide range of participants’ age and health conditions [5,6,7]. Therefore, there is a topmost need for more research in each population and age group with possibly uniform technologies and standardized diagnostic criteria.

Among several available technologies for body composition assessment, dual-energy X-ray absorptiometry (DXA), enabling synchronized assessment of all three body composition components, was utilized most frequently [13]. However, even in these studies, there were some inconsistencies. The criteria to diagnose osteopenia and osteoporosis were uniform based on T-score (less than −1 and −2.5, respectively), but in some studies, only cases with osteoporosis were considered, while in others, both osteopenic and osteoporotic cases were included. Regarding the diagnosis of sarcopenia, usually, the appendicular skeletal muscle adjusted for height (ASM/Ht^2^) was taken, but in some studies, functional performance measures (e.g., grip strength, gait speed, and/or balance) were applied either alone or complemented with ASM/Ht^2^ to derive a sarcopenia/dynapenia diagnosis. The cutoffs for body fat varied, ranging from 30 to 40% for women and 25 to 28% for men. Additionally, in some instances, the total body fat was used, while in others, only the visceral fat was used. Still, in some studies, the body mass index (BMI) and waist circumferences were employed either solely or in addition to the body fat percentage to determine the overweight/obesity status. Another typically utilized technology to measure body composition was bioelectrical impedance analysis (BIA) for lean and fat and ultrasound devices for bone tissue assessment. The results from these studies also varied depending on what kind of instrument was in question, e.g., single- or multi-frequency analyzers, the latter being shown to be more reliable [14]. Some of the newer BIA analyzers also render the values for total bone mass (in addition to lean and fat), making the results more reliable and measurements more convenient for both participants and researchers.

Regarding the geographic region and populations, the majority of the studies were conducted in Eastern Asia, specifically China, Japan, and Korea, utilizing large databases and enabling robust analyses [15,16,17,18,19,20]. Fewer studies were conducted on the Caucasian population in Europe and/or North America [6,21,22], on populations in South America [23], and none on black populations. Additionally, the majority of participants were older women (>60 years), and many also had some lingering chronic diseases. 

Our objective was to use the most recent criteria for the OSA diagnosis [6] and calculate its prevalence in a large population of apparently healthy individuals from the same geographic region and ethnicity but in different age categories. Such an approach aligns with the main principles of personalized medicine, where similar individual characteristics within the group (e.g., regional, ethnic, phenotypic, environmental, and/or societal) are applied to obtain important health information and design possible prevention/management strategies. Another objective was to examine and possibly rationalize the most obvious reasons for the noted discrepancies in OSA prevalence.

## 2. Materials and Methods

### 2.1. Participants

The participants were part of a large multicenter cross-sectional study conducted in Italy between 2010 and 2014, which included Caucasian women and men between 18 and 90 years of age who visited 37 Italian general medical practices. The aim of this study was to assess bone and body composition, as well as functional status, in the Italian general population. 

All participants signed a written consent form and completed a medical history questionnaire. This study was approved in Italy by the Consortium for the Science and Technology Research AREA, Trieste, Italy, and conformed to the standards set by the Declaration of Helsinki. Exclusion criteria were a history of chronic disease (severe mental/psychiatric disorder, epilepsy, cancer, rheumatic disease, atopic dermatitis, peripheral neuropathy, myopathy, stroke, Parkinson’s disease, severe dementia, significant cognitive impairment), recent fractures (<12 months), heavy alcohol consumption, use of bone active therapies (steroids, bisphosphonates, calcium, vitamin D, or hormones), as well as established or suspected pregnancy and the presence of metal prostheses or implanted electronic devices (e.g., heart pacemakers). In this study, we retrospectively analyzed the part of the collected data from the original study. This post hoc analysis was approved by the Ethics Committee of the Institute for Medical Research and Occupational Medicine (No. 100-21/18-10) in Croatia.

### 2.2. Anthropometric and Bioimpedance Measurements

Body height (cm) and weight (kg) were measured with standard methods, and body mass index (BMI; kg/m^2^) was calculated. Body composition was assessed with a bioelectrical impedance device BIA-ACC (BioTekna^®^, Marcon–Venice, Italy). All medical devices by BioTekna^®^ are non-invasive medical devices used for screening, diagnostic, and monitoring purposes and have been approved and used in the European Union since 2004. We also received approval from the Ethics Committee of the Institute for Medical Research and Occupational Medicine (No. 100-21/18-10) for the use of the BIA-ACC BioTekna^®^ device for research/scientific purposes. Besides measuring soft (lean and fat) tissues and body water, the BIA-ACC device enables the measurement of total bone mass (kg), providing T-scores for normal, osteopenic, and osteoporotic bones. The device was validated against DXA technology (the gold standard for body composition assessment) [24].

The measurements were performed while participants were in a supine position with legs slightly spread and arms not touching the body. Two electrodes were placed on the right hand (metacarpal and wrist areas) and two on the right leg (metatarsal and ankle areas). The measured parameters were: fat-free mass (FFM; as % of body weight); total bone mass (BM; kg) yielding T-score; skeletal muscle mass (SM; as % of FFM) yielding S-score; fat mass (FM; as % of body weight); and intramuscular adipose tissue (IMAT; as % of body weight). Osteopenia and osteoporosis were diagnosed based on the T-scores (osteopenia from −1 to −2.5 and osteoporosis below −2.5). Sarcopenia was diagnosed based on the S-score of ≤−1.0 and obesity/adiposity based on total fat mass ≥25% of total body weight for men and ≥32% for women [6]. The simultaneous presence of osteopenia/osteoporosis, sarcopenia, and obesity/adiposity was a condition for OSA syndrome diagnosis. 

### 2.3. Statistical Analysis

The results are presented as mean and standard deviation for continuous variables and as percentages for categorical variables. The normality of the distribution was tested using the Kolmogorov–Smirnov test. Since the majority of variables were not normally distributed, the differences between men and women were tested with the Mann–Whitney U test for continuous variables and with the chi-square test for categorical variables. ANOVA with a post hoc Tukey test was used to analyze the differences in body composition and OSA prevalence among participants stratified into age groups. A statistical significance was defined at the *p* < 0.05 level.

## 3. Results

A total of 9719 participants (*n* = 6412 women, 65.9%) were included. The mean age of all participants was 47.7 years. All body composition parameters, except FM%, were significantly higher in men than in women (*p* < 0.001) (Table 1). According to BMI, more than half of women (55.2%) were in the category of normal body weight, while more than half of men were overweight or obese (57.9%). Significantly more women than men were undernourished (BMI < 18.5 kg/m^2^, 3.5% vs. 0.6%, respectively; *p* < 0.001). Mean values of FM% and IMAT% were higher in both women and men, while mean T-score values were lower in women with respect to the normal reference. 

Osteoporosis was detected in 3.9% of women and none in men, and osteopenia in 58.5% of women and 20.4% of men (*p* < 0.001). Significantly more women (54.9%) than men (28.1%) had sarcopenia (*p* < 0.001). Interestingly, obesity, determined by increased FM%, was detected in 65.1% of women and 86.1% of men (*p* < 0.001). In all age groups, men had a significantly higher prevalence of obesity than women and, at the same time, a significantly lower prevalence of sarcopenia and osteopenia/osteoporosis (Table 2). The exception was the oldest age group, where the difference was significant only for obesity. The prevalence of overweight/obesity according to BMI (≥25.0 kg/m^2^) was lower than that according to FM% in all age groups and was also significantly higher in men than in women, except in the oldest age group. 

**Figure 1:**
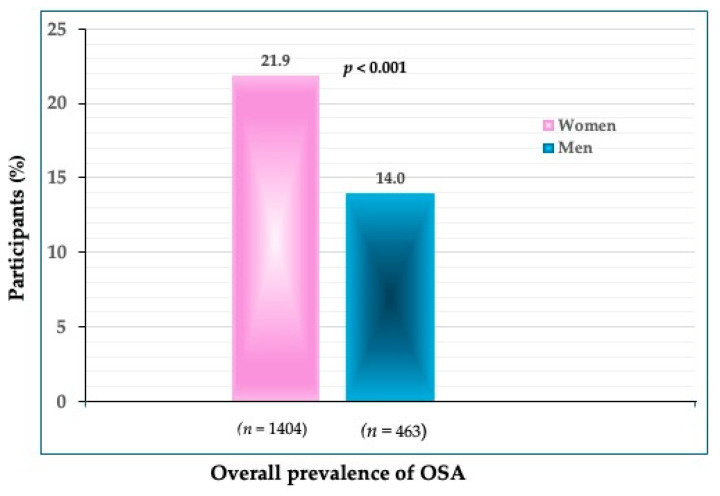
The overall prevalence of OSA in women and men (*n* shows the number of participants in each group).

**Figure 2:**
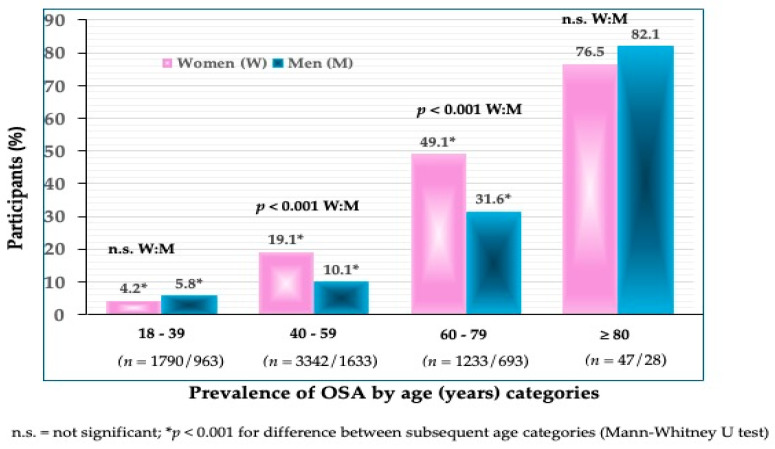
Prevalence of OSA according to age categories in women and men (*n* shows the number of participants in each group).

The prevalence of OSA in all participants was 21.9% in women and 14.0% in men (*p* < 0.001) (Figure 1). The prevalence of OSA significantly increased in every subsequent age group in both women and men (Figure 2). In the youngest age group, the difference was not significant between women and men (4.2 vs. 5.8%). In the age groups between 40 and 59 years, the prevalence was significantly higher in women than in men (19.1 vs. 10.1%) (*p* < 0.001), and so was in those between 60 and 79 years (49.1 vs. 31.6%). As expected, the highest prevalence of OSA was in the oldest participants (>80 years), in both women (76.7%) and men (82.1%). Interestingly, in that age group, the prevalence of OSA was higher in men than in women, although not significantly.

Participants with OSA were significantly older than those without (*p* < 0.001) (Table 3) and, as expected, had significantly higher FM% (only women), IMAT%, and significantly lower T-score and S-score than those without OSA (*p* < 0.001). However, women and men with OSA had significantly lower BMI compared to those without OSA (*p* = 0.001 for women; *p* < 0.001 for men).

When analyzing the difference in body composition according to age groups in participants with OSA, there was a significantly higher FM% and IMAT% in each older age group, both in women and men (*p* < 0.001) (Table 4). The exception was FM% in the two oldest age groups, where the difference was not significant and the BMI was lower. Notably, BMI decreased in the oldest age group in women and men, although that change was not significant. Significantly lower T-scores and S-scores in each older age group were found in women (*p* < 0.001) and men (*p* = 0.024, 0.042, and < 0.001). 

## 4. Discussion

To our knowledge, this is one of just a few studies reporting the prevalence of OSA in community-dwelling, apparently healthy Caucasian participants spanning across a wide age range and of the same ethnic origin. The overall prevalence of OSA was 21.9% in women and 14.0% in men, with a significantly higher prevalence in older compared to younger age groups in both women and men (Figure 1 and Figure 2). 

Because there was such a variety in the design and geographic regions of studies investigating OSA syndrome, it was hard to find a close comparison to our study. Nevertheless, we addressed some studies with the most extreme results on OSA prevalence and provided possible explanations. For example, with regard to the age of participants, one of the studies comprising adults of a wide age range (21–90 years) was performed on 463 community-dwelling adults (~58% females) from Yishun, Singapore [25]. The participants were multiracial, although mostly Chinese residents, and DXA was utilized for body composition assessment. The prevalence of OSA depended on each of the criteria the authors used to determine sarcopenia and/or obesity. Sarcopenia was diagnosed either by low muscle mass (by DXA) or following the Asian Working Group for Sarcopenia (AWGS) 2019 criteria [26]. Similarly, obesity was determined either by FM% or fat mass index (the ratio of fat mass to fat-free mass). In any case, the prevalence was substantially lower than in our study, both for younger and older participants. The population-adjusted prevalence ranged from 0–1% in the group under 60 years, to 2.8–4.1% in the 60–65 years group, to 5.3–11.0% in the group >75 years (always higher in women). In another study conducted on Chinese women (*n* = 2315) of several ethnic minorities and of a wide age range (20–90 years), the OSA prevalence was 0.4% in younger (<60 years) and 11.5% in older (≥60 years) women, with the higher prevalence associated with dyslipidemia [27]. The measurements in that study were performed by BIA for lean and fat and by heel ultrasound for bone tissue; thus, the prevalence could have been affected either by different ethnicities or different technologies used to assess soft and bone tissues. In another study on Chinese participants, Nie et al. investigated OSA and systemic inflammation in 648 adults (64.2% females, mean age 67.2 years), with a reported total prevalence of 16.5%. Importantly, the OSA presence was associated with a higher systemic inflammatory index [20]. All components of body composition were assessed with DXA, providing more convenient and possibly more reliable measurements than when different technologies were used for bone and soft tissue assessment.

There were a few studies conducted in South Korea utilizing the Korean National Health and Nutrition Examination Survey (KNHANES) data, thus rendering the analysis of a large number of community-dwelling individuals. They were all measured by DXA for body composition assessment. For example, in the study by Lee et al. [16], examining the OSA in relation to insulin resistance in 4500 individuals (54% females), although all body compartments were assessed by DXA, obesity was determined by both BMI and FM%, the latter rendering higher obesity and OSA prevalence (5.8% and 7.2% for men and women, respectively). The prevalence was <1% when obesity was determined based on BMI, showing quite a variation in the results. Although the classification of obesity based on BMI is widely used in clinical practice, its limitations for these purposes have been addressed [28]. Reports from two studies [17,18] also utilizing KNHANES data revealed quite a wide range in OSA prevalence in women and men older than 60 years. It was 10.9% in both women and men [18], and ~92% in women and ~33% in men [17]. Of note is that in these studies, the higher prevalence of OSA was associated with lower calcium and protein intake, respectively. Both of these studies were conducted by the same group of researchers, utilizing KNHANES 2008–2009 [17] and KNHANES 2008–2011 [18], yet resulted in such a different prevalence. It is hard to speculate about all the reasons, but the most obvious are the different cutoffs used for obesity and the lack of adjustment for height when calculating sarcopenia via ASM [17]. In another study [29] conducted in the Korean population (>60 years), using the DXA technology, the OSA prevalence was ~40% and ~28% in women and men, respectively, and was associated with lower serum vitamin D. This prevalence was close to that of our participant in the 60–79 age category (Figure 2). 

Su et al., utilizing the Taiwanese Health Management Center database, investigated the relationship between OSA components and metabolic syndrome in 2291 participants (42% females) above 50 years and reported a total OSA prevalence of 4.7%, but 6% in those with metabolic syndrome [15]. Both DXA and dynamometers (for grip strength) were used for sarcopenia detection, which could have created some discrepancies with other studies and a possible lower incidence of sarcopenia when grip strength was applied in diagnosis. In a Japanese study investigating the association between OSA and non-alcohol fatty liver disease (NAFLD) in 614 patients (mean age 66.7 years, 55% women), the prevalence of OSA was also low (6% and 1% for women and men, respectively) and was significantly positively associated with NAFLD in women [19]. This is a surprisingly low prevalence, considering that the participants were patients with NAFLD. However, the identification of osteopenia/osteoporosis followed the Japanese criteria, using the young adults’ mean bone mineral density values (%YAM) cutoffs, where values between 70% and 80% indicate osteopenia and those below 70% osteoporosis [30]. The diagnosis of sarcopenia was based on AWGS criteria [26], which are not necessarily always used in studies involving Asian populations, thus precluding comparisons among those population groups. Notably, while the OSA prevalence varied and was generally lower than in our study, in most cases, the presence of OSA or its higher prevalence was associated with poor diet or some chronic conditions. 

Studies of the Caucasian population, especially in Europe and the United States, are rare. A post hoc analysis from the study in the United States conducted on ~500 women with overweight/obesity across a wide age range revealed that 25% had osteosarcopenic obesity [6]. Regarding the Mexican population, the prevalence reported from the study conducted on middle-aged to older women was ~19% [23]. In both studies, DXA was utilized for body composition assessment, and notably, the presence of OSA was associated with lower functionality measures. In the study conducted in Iran [22], the total prevalence in 2339 participants over 65 years of age (51% women) was 19.8%, with 22.1% in females and 17.4% in males. The results from both Szlejf et al. [23] and Ahmadinezhad et al. [22] are comparable to the overall prevalence of 17.3% in our participants aged 40–80 years, although DXA technology was used in those studies as opposed to BIA utilized in our study. In our oldest participants (>80 years), the prevalence in women (76.5%) was similar to that from the study conducted in older nursing home residents (70.8%) [31]. However, in the oldest men in the current study, the prevalence was higher compared to men of similar age in the nursing home study (82.1% vs. 47.8%). The most noticeable difference between the results of these studies and our results is the higher prevalence of OSA in older men in the current study, which exceeded that in women of the same age, although not significantly. A possible reason could be the existence of more health problems in older men or a smaller number of participants in the oldest age groups compared to those in the younger age groups. However, since we applied non-parametric statistical methods, we believe that the results of prevalence are comparable between all age groups in our study. 

Overall, the prevalence of OSA in our participants was higher than in most other studies discussed, except for the studies conducted in South Korea [17,18], which are hard to reconcile due to the extremely high discrepancies in the prevalence. The majority of these studies have been conducted in Japanese and Chinese populations, the latter also among different ethnicities, but with a large number of participants. It has already been established that Asian populations have a lower prevalence of both osteopenia/osteoporosis and sarcopenia, as well as obesity. The results of the meta-analysis by continents from 2021 showed the prevalence of osteoporosis at 16.7% in Asia and 18.6% in Europe [32]. Another meta-analysis from 2021 reported the global prevalence of sarcopenia, and according to the AWGS definition, 15% of the Asian population and 33% of Europeans had sarcopenia [33]. Lower obesity rates are also characteristic of Asians compared to European and/or American populations. When any one of the OSA components (osteoporosis, sarcopenia, and/or obesity) has a lower population prevalence, the prevalence of OSA will be lower as well. In addition, although considered the most accurate method for assessing body composition, DXA can estimate only about 75% of appendicular skeletal muscle mass [34], further indicating that each method has limitations in the estimation of body composition and ultimately contributes to differences in OSA prevalence. Therefore, in addition to different technologies and cutoff criteria, we may assume that there are ethnic variations in the prevalence of OSA that warrant further investigation. Additionally, the different prevalences of individual components of the OSA syndrome also result in an uneven global prevalence of OSA, as clearly reflected in this brief overview. 

There are just a few studies on OSA in which the same bioimpedance device (BIA-ACC, BioTekna) as in the current study was used [21,31,35], although this device has advantages to other BIA instruments as it captures the total bone mass in addition to lean and fat tissues. Among these studies, Stefanaki and colleagues [21] were the first ones to detect the presence of the OSA phenotype (although the prevalence was not reported) in healthy, young Italian adults (*n* = 2551, mean age 19.5 years) and relate it to elevated inflammatory markers. Similarly, the results from our recent studies in nursing home residents [31,35], where the OSA prevalence was >50% (comparable to the current study in older age groups), revealed a significant association of OSA with higher extracellular water (indicating a higher inflammatory state) and lower phase angle (indicating lower cell integrity). Of note is also that our participants, especially women below 40 years, had a relatively high prevalence of low muscle mass (42.0% in women and 20.8% in men) and low bone mass, primarily osteopenia (52.0% in women and 14.2% in men; Table 2). 

Although men globally have a higher prevalence of sarcopenia than women, especially in older age, we believe that such differences are not so uniform. There are studies in which the prevalence in women is higher than in men [36,37] or is equal [38]. We believe that the different prevalence in men and women is partly the result of methodological differences in the diagnosis of sarcopenia. Unfortunately, there is no consensus on sarcopenia diagnosis [39]. Such a consensus exists for osteopenia/osteoporosis to allow for accurate comparisons. In general, men had a higher prevalence of sarcopenia using the EWGSOP (European Working Group on Sarcopenia in Older People) criteria, while it was higher in women using the criteria of the International Working Group on Sarcopenia [33]. In our study, the increase in prevalence of both osteopenia and sarcopenia with age was consistent in women, while the rate of increase was more noticeable after the age of 60 years in men. This was expected since osteoporosis starts earlier and worsens faster in women because of midlife hormonal shifts. Later in life, both sexes lose bone/muscle at about the same rate [39,40]. The prevalence of overweight/obesity (defined either by BMI or FM%) in men under the age of 40 from our study was significantly higher than in women, while in older age, that difference became smaller and finally equalized in the oldest age group (Table 2). The sex differences in adiposity are driven by gonadal hormones. Estrogen generally provides a protective effect in females by increasing energy expenditure and protecting against weight gain [41]. The natural cessation of estradiol synthesis and the lack of estrogen after menopause result in more fat tissue. On the other hand, aging men have declining levels of testosterone throughout their lives, resulting in an increase in fat mass and decreased insulin sensitivity [42]. 

As expected and by definition, participants with OSA had inferior all three body composition components (lower bone and muscle and higher fat) compared to those without OSA (Table 3). Interestingly, both women and men with OSA had a lower BMI but a higher FM% compared to those without OSA. Such an incongruity was shown in another study [11], where the prevalence of obesity (and OSA) determined based on FM% was significantly higher than when determined based on BMI. This indicates that OSA is a separate disorder that can exist regardless of physiological fluctuations in weight and BMI, and that infiltrated fat (not captured by BMI) may play a more important role [4,5] (it again points to the inadequacy of BMI for determining overweight/obesity [5,39]). In this context and highlighting the importance of fat locations within the body, two phenotypes (based on fat location) in older subjects (predominantly women) were identified: osteosarcopenic visceral adiposity and osteosarcopenic subcutaneous adiposity [43]. DXA measurements of the android region were initially used for the detection of android fat and then transformed and analyzed by computed tomography to determine the visceral/subcutaneous adipose tissue ratio. The ratios above and below 1 distinguished between visceral and subcutaneous adiposity, respectively, and the subsequent identification of osteosarcopenic visceral and osteosarcopenic subcutaneous adiposity. Importantly, the results revealed a higher risk of fracture (by FRAX assessment), a higher inflammatory state, and an altered metabolic profile in participants with osteosarcopenic visceral adiposity compared to those with osteosarcopenic subcutaneous adiposity. Although this study had several limitations (e.g., a small sample size as the participants were stratified into several cohorts), it was the first to distinguish between two OSA phenotypes by adipose tissue location. More studies like this could lead to the establishment of cutoffs for identifying osteosarcopenic visceral adiposity and its association with other chronic conditions.

The advantage of our study is that it included a large number of participants of all age groups with similar geographic and sociodemographic influences, providing a more homogenous sample and, thus, more robust results for each particular age group. The results also revealed the tendency for a change in body composition from young to very old age, observed separately in men and women. Although strict exclusion criteria eliminated many participants and improved the homogeneity of our sample, we did not have information about other lifestyle factors such as diet and physical activity to account for. Another limitation of this study is the diagnosis of sarcopenia based on muscle/lean mass alone. The prevalence could have been somewhat lower if muscle strength were also considered (for instance, by measurement of handgrip strength), especially in younger participants. However, there is still no consensus on whether to use the full diagnostic algorithm (e.g., muscle mass, strength, and function) or just a low muscle mass criterion [39]. Many studies on OSA, in addition to those discussed in this paper, also based the diagnosis of sarcopenia only on muscle mass [44,45], making our study comparable regarding sarcopenia detection. 

## 5. Conclusions and Implications for Future Research

The overall prevalence of OSA among community-dwelling healthy Caucasian adults ranging in age from 18 to 90 years was 21.9% in women and 14.0% in men. It varied by age and, as expected, increased significantly with age in both women and men. Surprisingly, a relatively higher prevalence of sarcopenia and osteopenia was noticeable in younger women and obesity in younger men. The snapshot of the literature revealed a wide range of OSA prevalence, but generally lower in East Asian populations, implying the existence of ethnic specificity. Additionally, most studies reported a positive association between OSA and/or its higher prevalence in some chronic conditions; however, it is not known for sure whether these relationships are bidirectional. 

Considering that the OSA syndrome is a major body composition disorder exposing individuals to a greater risk for various diseases (or resulting from them) and is an important public health issue, the lack of standardized and consolidated criteria for its diagnosis is the biggest obstacle in identifying individuals with OSA and thus precluding rational and practical comparisons across studies. To obtain standardized criteria, we recently suggested some approaches and indications for future studies [7,8]. Briefly:

- While the majority of the studies were conducted in older women (either White or Asian), equally important would be the studies in younger individuals, as the evidence from the current study and earlier work [21] identified OSA phenotypes in healthy, young, obese individuals.
  ○ Therefore, both men and women of different ages and races/ethnicities (there are no studies on African Americans), as well as critical populations (e.g., institutionalized individuals), would provide for better diagnostic criteria.
    ▪ For example, a close approximation of its prevalence in the general population could be obtained from large population databases like NHANES, KNHANES, or the UK Biobank, which comprise relatively healthy participants of a wide age range and the same regional and environmental influences; however, the ethnic differences must be accounted for.
- Body composition could be measured by various devices (e.g., DXA, BIA, ultrasound for bones, BMI for obesity—not recommended); however, the prevalence of OSA should be compared among studies that used the same/similar technology.
  ○ Furthermore, to differentiate the OSA from other body composition impairments, the comparison should be made with those having osteopenia/osteoporosis, osteopenic adiposity, sarcopenia, sarcopenic adiposity, or osteopenic sarcopenia, or adiposity-alone, and most importantly with those of normal body composition parameters. This will provide more insight into OSA and its association with other health issues.
- The development of biomarkers for each tissue and their combination could also help in the identification of OSA; however, the investigation of these biomarkers in the context of OSA syndrome is still in the early stages.
  ○ Some pilot studies specified the combination of increased levels of serum sclerostin (a bone resorption marker), skeletal muscle troponin (a muscle breakdown marker), leptin (an indicator of higher adiposity), and an inferior lipid profile as possible markers for OSA. However, some fine-tuning and more studies are necessary. In this context, a series of omics will need to be determined to serve as potential biomarkers.
- Additionally, given the fast genomic developments (sequencing and molecular drug exploitation), “the precision medicine concepts can also be utilized to outline OSA using multiple data sources, from genomics to digital health metrics to artificial intelligence, to facilitate individualized yet “evidence-based” decisions regarding diagnostic and therapeutic approaches. In this way, therapeutics can be centered toward patients based on their molecular presentation rather than grouping them into broad categories with a “one-size-fits-all” approach” citation from [7].

Overall, a detailed assessment of body composition in all age groups, but especially in the elderly, is important for building a personalized preventive strategy for OSA and related disorders and, if in place, may ensure adequate and timely treatment and management approaches. Additionally, the relatively high prevalence of OSA in younger age groups noted in our study indicates that the particularly important target group for early detection of OSA is apparently healthy younger individuals with overweight and/or obesity (a rapidly growing population segment). This is a group where the simultaneous occurrence of osteopenia and sarcopenia, leading to the progression to OSA, may remain undiagnosed for a long time and eventually result in unfavorable clinical outcomes.

## Figures and Tables

**Table 1:** Age, anthropometry, and body composition in women and men (mean ± SD), with reference values.

Variables	Women (*n* = 6412)	Men (*n* = 3307)	*p* *	Reference Values
Age (y)	47.6 ± 13.3	47.8 ± 14.1	0.412	
Weight (cm)	66.7 ± 14.4	82.9 ± 15.1	<0.001	
Height (kg)	163.1 ± 6.6	176.4 ± 7.0	<0.001	
BMI (kg/m^2^)	25.1 ± 5.4	26.6 ± 4.6	<0.001	18–24.9
T–score	−1.1 ± 0.8	−0.3 ± 0.7	<0.001	>−1.0
S–score	−0.9 ± 1.4	−0.1 ± 1.2	<0.001	>−1.0
FM (%)	33.2 ± 8.6	32.5 ± 7.3	<0.001	12–31.9% W 7–24.9% M
IMAT (%)	2.0 ± 0.5	2.2 ± 0.4	<0.001	<2.0%

BMI = body mass index; FM = fat mass; IMAT = intramuscular adipose tissue; W = women; M = men. * Mann–Whitney U test (women–men).

**Table 2:** Prevalence (%) of osteopenia/osteoporosis, sarcopenia, and obesity according to age groups in women and men.

	18–39 (y)		40–59 (y)		60–79 (y)		≥80 (y)	
Variables	W *n* = 1790	M *n* = 953	W *n* = 3342	M *n* = 1633	W *n* = 1233	M *n* = 693	W *n* = 47	M *n* = 28
Osteopenia/ osteoporosis	52.0 ^a^	14.2	62.9 ^a^	15.6	75.3 ^a^	37.9	89.3	82.1
Sarcopenia	42.0 ^a^	20.8	56.4 ^a^	23.1	67.9 ^a^	47.6	89.3	89.2
Obesity (based on FM%)	39.1 ^a^	72.2	56.4 ^a^	90.3	81.3 ^a^	94.5	87.2 ^b^	100.0
Obesity (based on BMI ≥ 25 kg/m^2^	39.2 ^a^	53.5	48.6 ^a^	72.8	61.5 ^a^	78.9	87.2	85.7

^a^ *p* < 0.001; ^b^ *p* = 0.048 (chi-square test: differences between women and men in each age group). W = women; M = men; FM = fat mass; BMI = body mass index.

**Table 3:** The difference in age and body composition between participants with and without OSA (mean ± SD).

	Age (y)	BMI (kg/m^2^)	FM (%)	IMAT (%)	S-Score	T-Score
Women						
With OSA (*n* = 1358)	58.1 ± 11.5 ^a^	24.6 ± 2.2 ^a^	36.7 ± 3.9 ^a^	2.4 ± 0.3 ^a^	−1.8 ± 0.6 ^a^	−1.7 ± 0.4 ^a^
Without OSA (*n* = 5054)	44.7 ± 12.5	25.2 ± 6.0	32.2 ± 9.3	1.9 ± 0.5	−0.6 ± 1.4	−0.9 ± 0.8
Men						
With OSA (*n* = 464)	58.2 ± 14.5 ^a^	24.0 ± 2.4 ^a^	33.0 ± 4.8	2.4 ± 0.3 ^a^	−1.6 ± 1.4 ^a^	−1.3 ± 0.3 ^a^
Without OSA (*n* = 2843)	46.1 ± 13.3	27.0 ± 4.7	32.5 ± 7.6	2.2 ± 0.4	−0.0 ± 1.1	−0.1 ± 0.6

^a^ *p* < 0.001; differences between participants with OSA and without OSA (Mann–Whitney test).

**Table 4:** The difference in body composition between age groups in women and men with OSA.

Age Group (y)	Age (y)	BMI (kg/m^2^)	FM%	IMAT%	S-Score	T-Score
Women						
18–39 (*n* = 1790)	31.1 ± 5.8 ^a^	24.1 ± 5.1 ^a^	29.9 ± 8.6 ^a^	1.6 ± 0.5 ^a^	−0.5 ± 1.2 ^a^	−0.8 ± 0.7 ^a^
40–59 (*n* = 3342)	49.1 ± 5.4 ^a^	25.0 ± 5.3 ^a^	33.2 ± 8.3 ^a^	2.0 ± 0.4 ^a^	−0.9 ± 1.4 ^a^	−1.1 ± 0.8 ^a^
60–79 (*n* = 1233)	66.2 ± 4.9 ^a^	26.6 ± 5.7	37.8 ± 7.4	2.5 ± 0.3 ^a^	−1.4 ± 1.5 ^a^	−1.5 ± 0.9 ^a^
≥80 (*n* = 47)	83.3 ± 2.8	25.5 ± 4.3	39.0 ± 6.8	2.8 ± 0.3	−2.6 ± 1.1	−2.2 ± 0.6
Men						
18–39 (*n* = 953)	30.6 ± 5.8 ^a^	25.4 ± 4.7 ^a^	29.1 ± 7.5 ^a^	1.8 ± 0.4 ^a^	0.0 ± 1.1 ^b^	−0.1 ± 0.7 ^c^
40–59 (*n* = 1633)	49.3 ± 5.5 ^a^	27.0 ± 4.5 ^d^	33.2 ± 6.8 ^a^	2.2 ± 0.3 ^a^	−0.1 ± 1.1 ^a^	−0.2 ± 0.7 ^a^
60–79 (*n* = 693)	66.7 ± 4.9 ^a^	27.3 ± 4.5	35.5 ± 6.3	2.6 ± 0.3 ^a^	−0.7 ± 1.6 ^a^	−0.7 ± 0.7 ^a^
≥80 (*n* = 28)	82.9 ± 3.4	26.4 ± 2.8	37.6 ± 4.5	2.9 ± 0.2	−1.7 ± 0.7	−1.3 ± 0.4

^a^ *p* < 0.001; ^b^ *p* = 0.004; ^c^ *p* = 0.024; ^d^ *p* = 0.042; differences between two adjacent age group (ANOVA).

## Data Availability

The raw data supporting the conclusions of this article will be made available by the authors upon request.
